# Laboratory evaluation of the efficacy and speed of kill of lotilaner (Credelio^TM^) against *Ixodes ricinus* ticks on cats

**DOI:** 10.1186/s13071-018-2968-4

**Published:** 2018-07-13

**Authors:** Daniela Cavalleri, Martin Murphy, Wolfgang Seewald, Jason Drake, Steve Nanchen

**Affiliations:** 1 Elanco Animal Health, Mattenstrasse 24a, 4058 Basel, Switzerland; 2 Elanco Animal Health, 2500 Innovation Way, Greenfield, IN 46140 USA

**Keywords:** Lotilaner, Credelio^TM^, Cat, Tick, *Ixodes ricinus*, Efficacy, Speed of kill, Safety

## Abstract

**Background:**

Lotilaner, approved for dogs as a chewable tablet formulation, has separately been developed for oral use in cats (Credelio^TM^ chewable tablets for cats), to meet the need for an easy to use, safe and rapidly effective parasiticide. It is a valid cat- and owner-friendly alternative to topical products. This manuscript describes three pivotal laboratory studies assessing the efficacy and speed of kill of lotilaner in cats against *Ixodes ricinus* ticks following a single oral administration, at a dose rate close to 6 mg/kg.

**Methods:**

In Studies 1 and 2, efficacy and safety were evaluated 48 h after treatment and post-treatment weekly infestations in 16 cats, against untreated controls, for 35 days. In Study 3, efficacy and safety were assessed in 8 lotilaner-treated cats until Day 35, before and after 24 h incubation of the female live ticks removed from the animals 12, 18 and 24 h after dosing and subsequent weekly infestations.

**Results:**

Efficacy was > 99% on days 23 and 37, and 100% on all other timepoints in Study 1. In Study 2 it was > 98% on Days 9 and 37, and 100% on all other days. In Study 3, on Day 0, lotilaner was > 90% efficacious, pre- and post-incubation at all time-points. On Day 7, at 12 hours after infestation, efficacy was 100%, pre- and post-incubation. On Day 14, there was a 66.5% reduction in geometric mean live tick counts in treated cats compared to controls, increasing, after incubation, to 94.4%. Afterwards, efficacy decreased below 90% while tick counts in the treated groups remained significantly lower compared to controls. At 18 hours, lotilaner was ≥ 90% efficacious through Day 37, increasing to 100% at 24 hours, on all study days, with the exception of Day 28 (98.9 and 99.1% pre- and post-incubation, respectively). There were no treatment-related adverse events.

**Conclusions:**

At the minimum dose rate of 6 mg/kg, lotilaner was efficacious against *I. ricinus* ticks. In addition, lotilaner was effective against this tick within 12 hours of treatment, reaching 100% efficacy within 24 hours. Lotilaner sustained a rapid kill of newly infesting *I. ricinus* through 35 days. By quickly killing ticks that infest cats, lotilaner has the potential to contribute to the reduction of tick-borne pathogens transmission.

**Electronic supplementary material:**

The online version of this article (10.1186/s13071-018-2968-4) contains supplementary material, which is available to authorized users.

## Background

Lotilaner is the latest member of the isoxazolines, the newest ectoparasiticide class of compounds marketed for companion animals. Formulated as chewable tablets, it was approved for the treatment of tick and flea infestations in dogs (Credelio^TM^ chewable tablets for dogs; Elanco Europe Ltd.) [1–3]. After oral administration to an infested animal, this broad-spectrum parasiticide inhibits the gamma/aminobutyric acid-gated chloride channels of the ticks and fleas, resulting in rapid death of the parasites [4–6].

At present, no isoxazoline-containing ectoparasiticide for oral administration is available for the treatment of tick and flea infestations in cats. Isoxazolines, fluralaner (Bravecto®spot-on solution for cats; MSD Animal Health, Madison, NJ, USA), and sarolaner in combination with selamectin, (Stronghold Plus, Zoetis Belgium SA, Louvain-la-Neuve, Belgium), are approved for cats as topical spot-on applications.

In a market research performed during the lotilaner development programme (unpublished data), cat owners commonly expressed clear, negative feelings related to the administration of topical spot-on products, considering them disruptive of the owner-animal bond. Many owners responded welcoming the idea of an easy-to-administer flavoured, chewable oral tick and flea parasiticide for cats. A small, flavoured, cat-friendly, oral tablet would therefore be a valuable novel product able to fill the gap in tick and flea treatment of cats.

In a number of pivotal field and laboratory studies, the efficacy and clinical safety of lotilaner (Credelio^TM^ chewable tablets for cats, Elanco) against fleas (*C. felis*) and ticks (*Ixodes ricinus*) for 1 month, following oral administration at the minimum dose rate of 6 mg/kg, was confirmed [7–9].

The safety of the product was assessed in a pivotal tolerance study in which Credelio^TM^ was shown to be safe in 8-week-old kittens, at dose rates up to 130 mg lotilaner/kg administered monthly, for 8 months [10].

This manuscript describes three pivotal laboratory studies assessing the efficacy of lotilaner (Credelio^TM^ chewable tablets for cats) in cats experimentally infested with adult *Ixodes ricinus* ticks. Studies 1 and 2 assessed the efficacy of the product following a single oral administration at a dose rate of at least 6 mg/kg and as close as possible to this target dose, for five weeks. Study 3 evaluated the speed of kill of lotilaner tablets against adult *I. ricinus* ticks, 12, 18 and 24 hours post-treatment and subsequent weekly infestations, following a single oral administration to cats, at the same dose rate.

## Methods

These pivotal studies were performed in two different laboratories, with a randomized, blinded, parallel-group design. All studies were performed under GCP standards [11] and in compliance with the EU and WAAVP Guidelines for the testing and evaluation of the efficacy of antiparasitic substances for the treatment and prevention of tick and flea infestation in dogs and cats [12–14].

In each study, one (Studies 1 and 2) or more (Study 3), groups of eight lotilaner-treated cats were compared, for efficacy and safety, to an equal number of untreated negative control groups of the same size, after infestation with laboratory-bred *I. ricinus* ticks.

### Animals

In Study 1, European domestic shorthair cats were included; European mixed breed cats were enrolled in Studies 2 and 3. To be included, cats had to be at least 12 months (Study 1) or 7 months-old (Studies 2 and 3) and to be acclimatized to the study housing and conditions; they had to be healthy and non-pregnant, have undergone a sufficient washout period after previous treatments with acaricidal products, and have had a female tick attachment rate of at least 25% from an infestation performed approximately 1 week before the day of study treatment. In order for the lotilaner dose rate to be as close as possible to the minimum target of 6 mg/kg, cat bodyweight had to be in the ranges of 3.10–4.00 kg, 4.60–6.00 kg and 6.20–8.00 kg.

In Studies 1 and 2, 22 healthy cats of both sexes were selected for the acclimatisation period. In Study 3, 58 cats were acclimatised. Sixteen cats fulfilling all the inclusion criteria, no exclusion criteria, and with the highest female tick attachment rates in the pre-treatment infestation suitability test, were included in either Study 1 or 2. Forty-eight cats meeting the same inclusion/exclusion criteria were included in Study 3.

Cats in Study 1 were housed in isolated rooms of equal size and with the same environmental conditions. Each room had two identical pens. Cats were housed in groups of three or four in each pen. After randomization, the eight cats of the same treatment group were housed in the same room (four cats per pen). Both sexes were allowed, as all males included in the study were neutered.

Studies 2 and 3 were performed at a different test site; in both studies, animals of the same sex were pair-housed within the same treatment group.

In all experiments, after each tick infestation (i.e. from the infestation until completion of the tick count), cats were housed in individual cages for 48 h (± 2 h), with the exception of the infestation on Day -2, after which, cats were individually housed for 96 h (± 2 h), until Day 2.

### Randomisation

Cats fulfilling all inclusion criteria, no exclusion criteria, with at least a 25% attachment rate (live, attached, female ticks) and having the highest tick counts were included in the studies. For the randomization, cats were rank-ordered from highest to lowest pre-treatment infestation test tick counts and randomly allocated within blocks of two (Studies 1 and 2) or six (Study 3), to treatment groups. Cats of the same treatment group (and sex in studies 2 and 3) were randomly allocated to housing pairs.

### Blinding

Blinding was accomplished by separation of function. Study personnel responsible for general health observations, clinical observations, physical examinations and tick counting were blinded throughout the study. Only the sponsor’s representative, statistician, investigator, monitor and individuals responsible for the Investigational Veterinary Product (IVP) administration were aware of the treatment allocation of each cat. All documents where the cats’ group allocation was visible were kept inaccessible to blinded personnel and cats’ allocation was identified *via* study room numbers during the study. Blinding was maintained throughout all studies.

### Tick infestation and counting

Cats were experimentally infested with 50 ± 2 viable, unfed, adult, uniform in age, *I. ricinus* ticks (sex ratio approximately 70% female: 30% male) during the acclimatization period (pre-treatment infestation test to assess tick viability and cats individual tick retention rate) and on Days -2, 7, 14, 21, 28 and 35. To help tick attachment, cats were infested under sedation, according to laboratory standard practice: in Study 1 with intramuscular injection of medetomidine hydrochloride at 0.02 mg/kg and butorphanol at 0.1 mg/kg. In Study 2, sedation with 0.08 mg/kg medetomidine hydrochloride by intramuscular injection, was reversed ~90 min later using atipamezole hydrochloride administered by intramuscular injection at a dose rate of 0.2 mg/kg. Following sedation, cats were placed inside infestation cages and ticks released on the animals.

Tick removal and counts were conducted on Days -14 or -5 (Study 1 and 2, respectively, suitability test), on Day 2 at 48 ± 2 h after treatment administration as well as on Days 9, 16, 23, 30 and 37 (at 48 ± 2 h post-infestation).

In Study 3, tick counts with removal were performed on all cats on Day - 6, 48 h post-infestation. Additional counts were performed 12, 18 or 24 h post-treatment (Groups 1 and 4, 2 and 5, 3 and 6, respectively) on Day 0 and after new infestations (Days 8, 15, 22, 29 and 36) (Table 1).

**Table 1:** Study 3 treatment groups and tick counts timings

Cat group no.	Treatment group	Time of tick counts (h)
1	Control	12
2	Control	18
3	Control	24
4	IVP	12
5	IVP	18
6	IVP	24

*Abbreviation*: *IVP* Investigational Veterinary Product

Tick counts were performed according to each test facility’s standard procedure. Briefly, the procedure mainly consisted of a thorough examination of all body areas by palpation with the fingers tips (thumb counting) first to locate and count the attached and free ticks. Since only female *Ixodes* spp. ticks attach to the host [15], male ticks were not counted and not considered in any efficacy and attachment rate assessments. Ticks were categorized as free or attached and as alive or dead. Ticks were considered alive if their legs reacted to a tactile or CO_2_ stimulus (exhaled air) and were considered dead if they did not. They were considered attached if the tick’s mouthpart was firmly attached to the cat’s skin and were considered free if not. After count and categorization, ticks were discarded, with the exception of Study 3, in which, after removal from the animals, live ticks (free and attached) were incubated into a controlled environment (at 26–29°C and 70–90% relative humidity) and their viability re-assessed after 24 h.

### Treatment

All cats in the treatment groups received a single oral administration of the IVP (lotilaner, minimum dose rate of 6 mg/kg and as close as possible to this dose) on Day 0. Mock dosing was performed for the untreated controls. Treatment was administered 30 ± 5 min following feeding. Lotilaner was available in two tablet sizes (final formulation) of 12 and 48 mg, respectively. Administration was based on Day -2 body weights (Table 2).

**Table 2:** Credelio^TM^ dose administered and corresponding exposure to lotilaner

Body weight range (kg)	No. of tablets strength 12 mg	No. of tablets strength 48 mg	Dose rate (mg/kg)
3.10–4.00	2	0	7.74–6.00
4.60–6.00	3	0	7.83–6.00
6.20–8.00	0	1	7.74–6.00

### Study assessments

#### Efficacy

In Studies 1 and 2, efficacy was defined as the ability of lotilaner tablets to reduce tick infestations (live female ticks only) on cats, 48 h after treatment and 48 h after each post-treatment infestation. In Study 3, efficacy was defined as the ability of lotilaner tablets to reduce the female live *I. ricinus* tick counts on the cats on Day 0, 12 h after treatment, on Day 1, 18 or 24 h after treatment, on Days 7, 14, 21, 28 or 35, 12 h after tick infestation and on Days 8, 15, 22, 29 or 36, 18 and 24 h after tick infestation. In this study, efficacy was calculated for each time-point both before and after 24 h incubation of the live female ticks collected from the cats.

Lotilaner was considered effective against *I. ricinus* if efficacy was > 90.0% (female live ticks only) in the treated group *versus* the untreated control group at the evaluation time-point, provided that the infestation was adequate in the corresponding control group (average female ticks attachment rate of at least 25%), and the difference in live female ticks counts between treated and control groups was statistically significant (*P* < 0.05) between the two groups, with a significant decrease in tick counts in the treated group compared with controls using ANOVA.

Efficacy was calculated based on the percent reduction in female live tick arithmetic or geometric mean counts in the treated group compared with those in the corresponding control group using the Abbott’s formula:

$$ \mathrm{Efficacy}\;\left(\%\right)=100\times \left(\mathrm{MC}-\mathrm{MT}\right)/\mathrm{MC} $$

where MC is the arithmetic or geometric mean number of female live ticks on cats in the untreated control group and MT is the arithmetic or geometric mean number of female live ticks on treated cats.

Geometric mean calculation involved using the logarithm of the tick count of each animal; when any tick count was equal to zero, one was added to the count and later subtracted from the resultant calculated geometric mean prior to calculating percent effectiveness.

#### Safety

The general health of all cats was observed by a trained technician once daily from the start of the acclimatization phase to the end of study, with the exception of the study days in which clinical observations were performed by a veterinarian (Day 0 both pre-treatment and 1, 6 and 8 h following treatment administration). Clinical observations included general health, behaviour and appetite. Thorough physical examinations were performed by a veterinarian during acclimatization, to evaluate whether cats’ health allowed for their inclusion in the study and at study completion. All cats were monitored for adverse events (AEs) and serious adverse events (SAEs) throughout the study. Bodyweight was measured in fasted animals on Days -16, -2, 19, 26, 33 and 40 in Study 1 and on Days -8, -2 and 37 in Studies 2 and 3.

#### Statistical analyses

All efficacy analyses were performed in the intent-to-treat (Study 1) or per-protocol analysis (Studies 2 and 3) sets, which included all randomized animals that received the IVP or were left untreated, or all treated and untreated animals with no major deviations, respectively. All animals that received treatment were included in safety analyses (i.e. bodyweight and adverse events).

Eight animals per group were considered an adequate sample size as a minimum of six subjects per group is recommended by the EMEA and the WAAVP guidelines [12, 14]. All hypotheses were tested at a two-sided 0.05 level of significance. All analyses were performed using the SAS/STAT® software.

To test the sensitivity of the model, an analysis of variance (ANOVA) using logarithmically transformed counts (live attached + live unattached female ticks) was performed for the treated and control groups. Models included fixed the “Treatment” effect and random “Block” effect.

The following hypotheses were tested for Studies 1 and 2. Separate calculations were performed for each time point:

H0: Treatment group count = Negative control group count

HA: Treatment group count ≠ Negative control group count

When at least one tick count was equal to zero, one was added to the count before logarithm calculation for every animal in every treatment group prior to performing the transformation. Because it was likely that the assumption of normal distribution of log-transformed tick count was not valid, non-parametric methods were additionally used to compare the two groups (Mann-Whitney U-test) only in Study 1.

In all studies, tick infestation was considered adequate at each time point when the group mean female tick retention rate on the control animals was at least 25%, in compliance with the EU guidelines [12, 13].

ANOVA was used to compare the treated group with the untreated controls, with a fixed effect of “Treatment Group” and random effect of “Block”.

In Study 3, tick count data before and after incubation on a given measurement day/hour were compared separately between each control and treated group using ANOVA and logarithmically transformed counts. The model included the fixed effects of “Time (prior or post)” and “Subject”.

Bodyweight changes were calculated as the change in baseline weight (weight closest to dosing) to weight at the end of the biological phase and analysed using an ANOVA (SAS PROC MIXED) with a fixed “Group,” “Treatment” and “Treatment Group” effect in Studies 1, 2 and 3, respectively, and a random “Block” effect in all three studies.

Adverse events were described and counted.

French translation of the Abstract is available in Additional file 1.

## Results

### Efficacy

#### Adequacy of infestation

An adequate female tick infestation rate of ≥ 25% in the control groups was achieved at each study time point in all three studies; mean retention rates ranged between 52.5–70.7% in Study 1, 47.9–60.7% in Study 2 and 37.5–50.4% both pre- and post-incubation in Study 3.

#### Calculated efficacy and difference in live tick counts between treatment groups

In Studies 1 and 2, the difference in live, female *I. ricinus* geometric mean tick counts between the treated and untreated groups was statistically significant at all assessment time-points, with significantly lower counts in the lotilaner-treated groups (*P* < 0.0001; all days) (Tables 3 and 4).

**Table 3:** Geometric (arithmetic) mean counts of live, female *I. ricinus* ticks and percent efficacy in Study 1 (*n* = 8)

Day	Untreated control		Lotilaner			Comparison	
	Mean	Range	Mean	Range	Efficacy (%)	*t*-value	*P*-value
2	23.09 (23.63)	15–29	0 (0)	0–0	100 (100)	*t*_(7)_ = 39.1	< 0.0001
9	21.86 (22.63)	13–30	0 (0)	0–0	100 (100)	*t*_(7)_ = 31.1	< 0.0001
16	21.82 (22.13)	17–29	0 (0)	0–0	100 (100)	*t*_(7)_ = 50.8	< 0.0001
23	18.19 (18.38)	14–24	0.09 (0.13)	0–1	99.5 (99.3)	*t*_(7)_ = 29.9	< 0.0001
30	24.51 (24.75)	19–29	0 (0)	0–0	100 (100)	*t*_(7)_ = 62.1	< 0.0001
37	19.23 (19.75)	12–26	0.09 (0.13)	0–1	99.5 (99.4)	*t*_(7)_ = 32.6	< 0.0001

**Table 4:** Geometric (arithmetic) mean counts of live, female *I. ricinus* ticks and percent efficacy in Study 2 (*n* = 8)

Day	Untreated control		Lotilaner			Comparison	
	Mean	Range	Mean	Range	Efficacy (%)	*t*-value	*P*-value
2	17.39 (17.63)	12–20	0 (0)	0–0	100 (100)	*t*_(7)_ = 46.5	< 0.0001
9	20.90 (21.25)	16–27	0.19 (0.38)	0–3	99.1 (98.2)	*t*_(7)_ = 16.2	< 0.0001
16	20.07 (20.50)	15–27	0 (0)	0–0	100 (100)	*t*_(7)_ = 40.3	< 0.0001
23	19.17 (19.63)	14–26	0 (0)	0–0	100 (100)	*t*_(7)_ = 37.4	< 0.0001
30	20.69 (21.25)	14–29	0 (0)	0–0	100 (100)	*t*_(7)_ = 35.5	< 0.0001
37	16.34 (16.75)	11–25	0.09 (0.13)	0–1	99.4 (99.3)	*t*_(7)_ = 28.7	< 0.0001

Efficacy was > 90% on all assessment days. In Study 1, the efficacy was > 99% on Days 23 and 37, and 100% on all other timepoints (Table 3).

In Study 2, the efficacy of lotilaner was > 98% on Day 9, > 99% on Day 37 and 100% on all other days (Table 4).

In Study 3 there was a statistically significant difference (*P* ≤ 0.0182) in female live tick counts, before or after incubation, between each treated group compared to the corresponding untreated one, at all time-points, with lower counts in the treated groups (Tables 5, 6, 7 and 8).

**Fig. 1:**
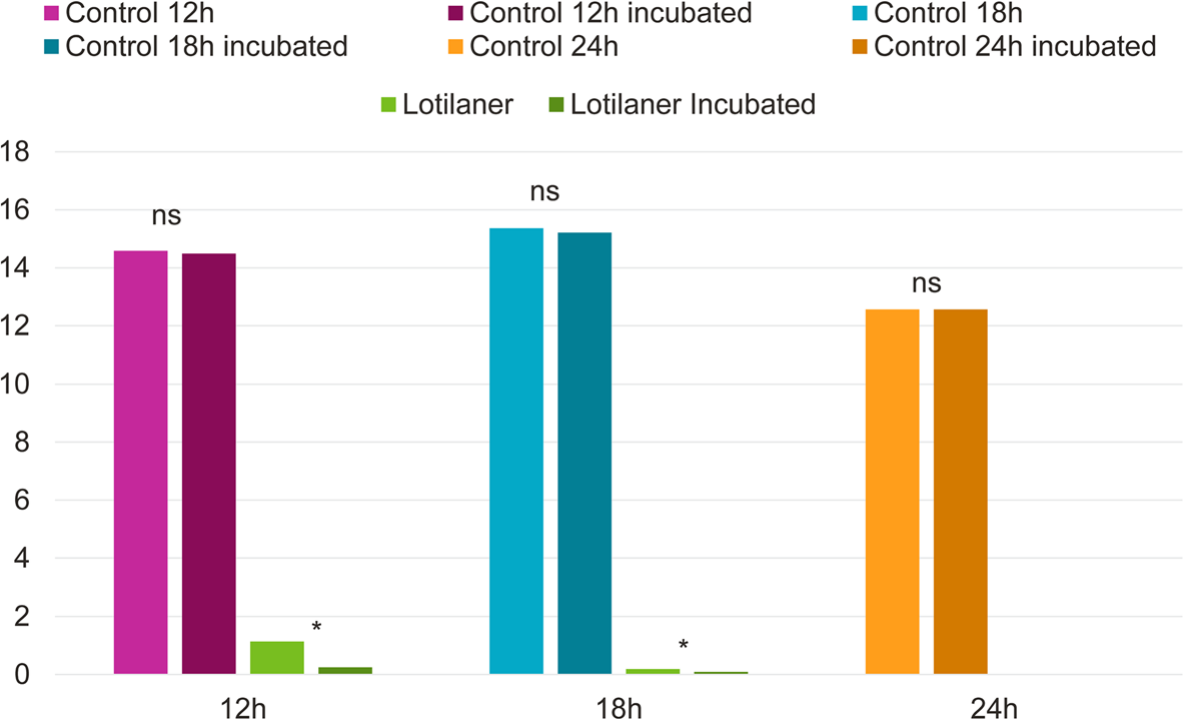
Geometric mean tick counts of the control and the lotilaner-treated groups before and after incubation on Day 0 at 12 h, 18 h and 24 h post-treatment. Within-group comparisons before and after incubation: ns, *P* > 0.05; *P < 0.05

**Fig. 2:**
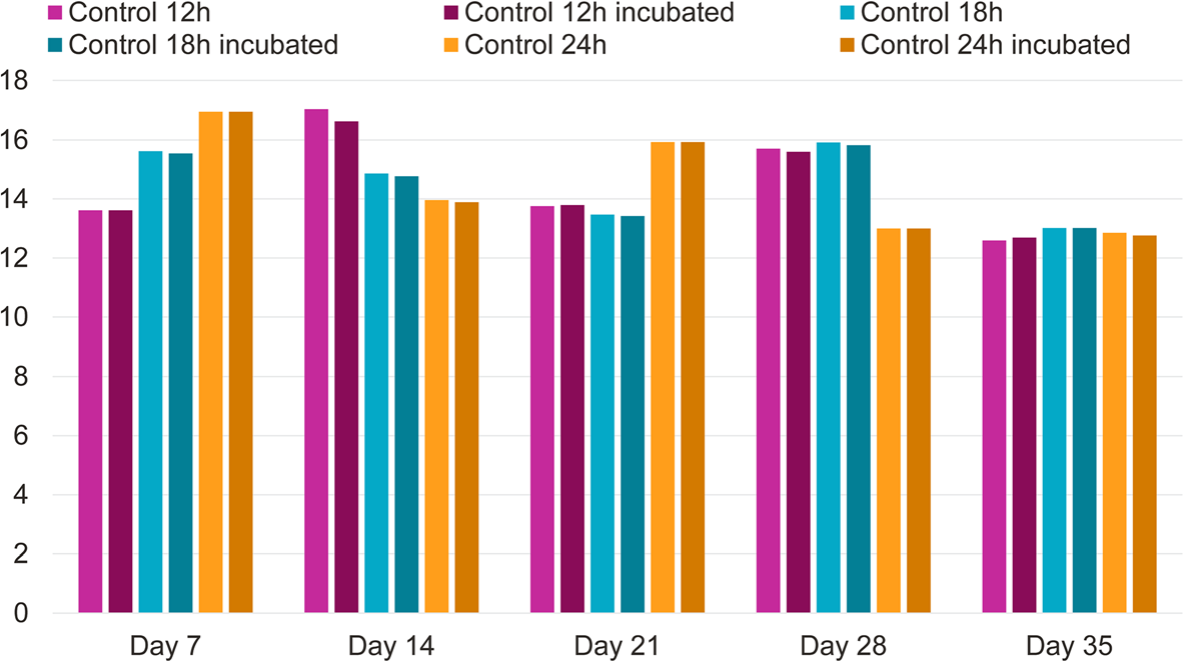
Geometric mean tick counts of the control groups before and after incubation on Days 7, 14, 21 and 28 at 12, 18 and 24 h post-infestation. *P* > 0.05 at all time points, for the within-group comparison before and after incubation

Mean live tick counts before and after incubation, were not significantly different, in the control groups at any time points (*P* > 0.05; Figs. 1 and 2).

In the treated group assessed at the 12 h time-point, post-incubation live tick counts were significantly lower (*P* < 0.05) compared to the pre-incubation counts on Days 0, 14, 21, 28 and 35. For the group assessed at the 18 h assessment point, post-incubation live tick counts were significantly lower (*P* < 0.05) on Days 15 and 22, while for the treated group assessed at the 24 h time-point, no significant difference between pre- and post-incubation live tick counts was observed.

**Fig. 3:**
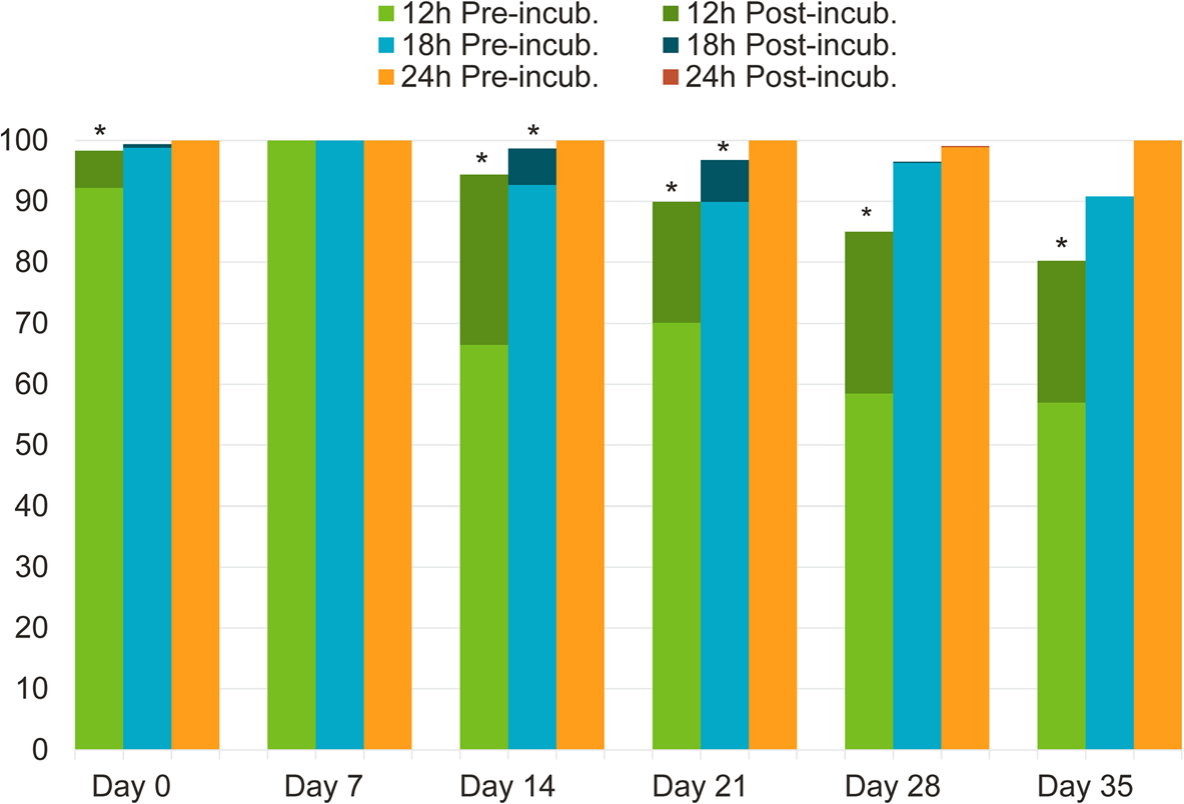
Percentage of efficacy (pre- and post-incubation) on Day 0 at 12, 18 and 24 h post-treatment and on Days 7, 14, 21, 28, 35 at 12, 18 and 24 h post-infestation. *P < 0.05 for the within-group comparison of live tick counts pre- and post-incubation

The efficacy of lotilaner after treatment administration (on a pre-existing infestation established on Day -2) met the > 90% threshold requirement laid out by the EU Guideline, at all time-points (Table 5 and Fig. 3).

**Table 5:** Geometric (arithmetic) mean counts of live, female *I. ricinus* ticks and percent efficacy of lotilaner against infestations present at the time of treatment

Time post-treatment	Untreated control		Lotilaner			Comparison	
	Mean	Range	Mean	Range	Efficacy (%)	*t*-value	*P*-value
12 h Pre-incubation	14.58 (15.63)	9–26	1.14 (1.75)	0–5	92.2 (88.8)	*t*_(7)_ = 7.6	0.0001
Post-incubation	14.48 (15.50)	9–26	0.25 (0.38)	0–2	98.3 (97.6)	*t*_(7)_ = 13.1	< 0.0001
18 h Pre-incubation	15.36 (16.75)	7–26	0.19 (0.25)	0–1	98.8 (98.5)	*t*_(7)_ = 14.4	< 0.0001
Post-incubation	15.21 (16.63)	7–26	0.09 (0.13)	0–1	99.4 (99.2)	*t*_(7)_ = 17.4	< 0.0001
24 h Pre-incubation	12.56 (13.13)	6–18	0 (0)	0–0	100 (100)	*t*_(7)_ = 22.8	< 0.0001
Post-incubation	12.56 (3.13)	6–18	0 (0)	0–0	100 (100)	*t*_(7)_ = 22.8	< 0.0001

At the 12 h post-challenge assessment, lotilaner efficacy was 100% (pre- and post-incubation) on Day 7. On Day 14, the efficacy was 66.5% and 94.4% pre-and post-incubation, respectively, subsequently decreasing to values below the 90% efficacy threshold (Fig. 3 and Table 6). At the 18 h assessment, lotilaner met the efficacy threshold through Day 37 (Fig. 3 and Table 7) and increased to 100% at the 24 h time-point, on all study days, except for Day 28 (> 98%) (Fig. 3 and Table 8).

**Table 6:** Geometric (arithmetic) mean counts of live, female *I. ricinus* ticks and percent efficacy of lotilaner, 12 hours after post-treatment infestations

Day^a^	Time-point	Untreated control		Lotilaner			Comparison	
		Mean	Range	Mean	Range	Efficacy (%)	*t*-value	*P*-value
7	Pre-incubation	13.62 (13.75)	11**–**17	0 (0)	0**–**0	100 (100)	*t*_(7)_ = 53.5	< 0.0001
	Post-incubation	13.62 (13.75)	11**–**17	0 (0)	0**–**0	100 (100)	*t*_(7)_ = 53.5	< 0.0001
14	Pre-incubation	17.02 (17.63)	12**–**28	5.71 (7.88)	1**–**18	66.5 (55.3)	*t*_(7)_ = 3.1	0.0182
	Post-incubation.	16.63 (17.13)	11**–**25	0.93 (1.38)	1**–**3	94.4 (92)	*t*_(7)_ = 8.3	< 0.0001
21	Pre-incubation	13.76 (14.13)	9**–**18	4.12 (5.00)	1**–**11	70.1 (64.6)	*t*_(7)_ = 4.5	0.0029
	Post-incubation.	13.79 (14.13)	9**–**18	1.39 (1.88)	0**–**5	89.9 (86.7)	*t*_(7)_ = 7.3	0.0002
28	Pre-incubation	15.70 (16.00)	12**–**21	6.52 (7.63)	2**–**15	58.5 (52.3)	*t*_(7)_ = 3.7	0.0072
	Post-incubation.	15.60 (15.88)	12**–**21	2.34 (2.75)	1**–**5	85 (82.7)	*t*_(7)_ = 8.1	< 0.0001
35	Pre-incubation	12.59 (14.63)	4**–**23	5.41 (7.38)	3**–**19	57 (49.6)	*t*_(7)_ = 3.2	0.0151
	Post-incubation	12.69 (14.50)	4**–**23	2.51 (3.38)	0**–**9	80.2 (86.7)	*t*_(7)_ = 5.0	0.0016

^a^Day of challenge

**Table 7:** Geometric (arithmetic) mean counts of live, female *I. ricinus* ticks and percent efficacy of lotilaner, 18 hours after post-treatment infestations

Day^a^	Time-point	Untreated control		Lotilaner			Comparison	
		Mean	Range	Mean	Range	Efficacy (%)	*t*-value	*P*-value
7	Pre-incubation	15.62 (17.13)	8–31	0 (0)	0–0	100 (100)	*t*_(7)_ = 18.0	< 0.0001
	Post-incubation	15.54 (17.00)	8–31	0 (0)	0–0	100 (100)	*t*_(7)_ = 18.2	< 0.0001
14	Pre-incubation	14.86 (17.25)	6–30	1.09 (1.88)	0–7	92.7 (89.1)	*t*_(7)_ = 6.2	0.0004
	Post-incubation.	14.77 (17.13)	6–30	0.19 (0.25)	0–1	98.7 (98.5)	*t*_(7)_ = 14.9	< 0.0001
21	Pre-incubation	13.47 (16.00)	3–31	1.36 (2.00)	0–5	89.9 (87.5)	*t*_(7)_ = 6.3	0.0004
	Post-incubation.	13.42 (15.88)	3–30	0.44 (0.63)	0–2	96.8 (96.1)	*t*_(7)_ = 10.0	< 0.0001
28	Pre-incubation	15.91 (17.00)	7–26	0.59 (1.38)	0–7	96.3 (91.9)	*t*_(6)_ = 6.6	0.0006
	Post-incubation.	15.82 (16.86)	7–25	0.40 (0.75)	0–4	97.5 (95.6)	*t*_(6)_ = 8.9	0.0001
35	Pre-incubation	13.01 (14.29)	8–25	1.20 (2.88)	0–10	90.8 (79.9)	*t*_(6)_ = 4.8	0.0032
	Post-incubation	13.01 (14.29)	8–24	1.20 (2.88)	0–10	90.8 (79.9)	*t*_(6)_ = 4.8	0.0032

^a^Day of challenge

**Table 8:** Geometric (arithmetic) mean counts of live, female *I. ricinus* ticks and % efficacy of lotilaner, 24 hours after post-treatment infestations

Day^a^	Time-point	Untreated control		Lotilaner			Comparison	
		Mean	Range	Mean	Range	Efficacy (%)	*t*-value	*P*-value
7	Pre-incubation	16.94 (17.50)	9–24	0 (0)	0–0	100 (100)	*t*_(7)_ = 29.5	< 0.0001
	Post-incubation	16.94 (17.50)	9–24	0 (0)	0–0	100 (100)	*t*_(7)_ = 29.5	< 0.0001
14	Pre-incubation	13.96 (15.25)	6–27	0 (0)	0–0	100 (100)	*t*_(7)_ = 17.3	< 0.0001
	Post-incubation.	13.89 (15.13)	6–26	0 (0)	0–0	100 (100)	*t*_(7)_ = 17.5	< 0.0001
21	Pre-incubation	15.93 (17.63)	5–29	0 (0)	0–0	100 (100)	*t*_(7)_ = 15.8	< 0.0001
	Post-incubation.	15.93 (17.63)	5–29	0 (0)	0–0	100 (100)	*t*_(7)_ = 15.8	< 0.0001
28	Pre-incubation	13.00 (13.50)	9–20	0.15 (0.25)	0–2	98.9 (98.1)	*t*_(7)_ = 14.8	< 0.0001
	Post-incubation.	13.00 (13.50)	9–20	0.09 (0.35)	0–0	99.1 (99.3)	*t*_(7)_ = 19.4	< 0.0001
35	Pre-incubation	12.85 (13.88)	6–24	0 (0)	0–0	100 (100)	*t*_(7)_ = 18.1	< 0.0001
	Post-incubation	12.76 (13.75)	6-24	0 (0)	0–0	100 (100)	*t*_(7)_ = 18.4	< 0.0001

^a^Day of challenge

Across all post-treatment challenges, few live ticks were detected on lotilaner-treated cats.

### Safety observations

Abnormal signs were reported for two control cats in Study 2 and for 22 and 19 treated and control cats, respectively, in Study 3. In Study 1, mild or moderate dermatitis (mainly described as crusts on the head region) most likely due to tick bite reactions, was reported for all cats. Body weight changes from baseline recorded during the studies were not statistically significant (*P* > 0.05). Weight loss in Study 1 and Study 3 was similar in all treatment groups and improved after increase of the daily ration. In Study 1, two and three episodes of loose stool in the IVP and untreated group, respectively, were reported, as well as one episode of mild vomiting in two IVP cats, 14 and 19 days after dosing, respectively. None of these observations was considered treatment-related.

## Discussion

Study 1 and 2 confirmed that the dose rate of 6 mg/kg selected in the dose determination study (undisclosed data) was sufficient to control *I. ricinus* infestation for a duration of one month with efficacy close to or at 100% to Day 35.

Study 3 demonstrated that *I. ricinus* ticks present on cats before lotilaner administration are killed at 12 hours after dosing. Tick incubation in an environment aimed at preserving their viability, showed that the product starts killing new ticks infesting cats after treatment as soon as 12 hours after infestation, reaching the 90% efficacy threshold at 18 hours and 100% killing at 24 hours (with the exclusion of the 28 days time-point, where efficacy was 98.9 and 99.1% pre- or post-incubation, respectively). The soundness of the experimental methodology was demonstrated by the absence of a statistically significant difference between pre- and post-incubation live tick numbers in the control groups (Figs. 1 and 2). This confirmed that untreated ticks were not affected by the incubation and that the significant decrease in live tick counts in the lotilaner groups post-incubation was a true treatment effect. The rapid onset of activity against ticks is consistent with lotilaner’s rapid speed of kill against fleas [7] and pharmacokinetics data showing that, in cats, lotilaner is readily absorbed and reaches peak blood concentrations within four hours [16].

The somewhat faster onset of activity of lotilaner immediately after dosing can be explained by the fact that ticks already present on the animals at the time of treatment are immediately exposed to its activity, with no time required to select the attachment spot and start feeding.

Adequate mean tick attachment rates in the control groups at all time points validated the cat infestation model and confirmed the validity of the calculated efficacy results. Efficacy and speed of kill were confirmed for 35 days in all studies, giving some flexibility to the 30 days administration schedule recommended on the product label.

No other oral isoxazoline is available for cats at the time this paper is being written. Lotilaner appears to act faster against ticks present on cats at the time of treatment than the other systemically acting isoxazolines available as topical solutions. Fluralaner and sarolaner labels indicate onsets of action of 48 and 24 hours, respectively, on both pre-existing and new infestations. Against new infestations, lotilaner, with its onset of action of 18–24 hours (depending on whether pre- or post-incubation data and geometric or arithmetic means are considered), throughout five weeks, appears to be faster than fluralaner while comparing favourably with sarolaner [17].

Our studies were performed in compliance with the EU Guidelines current at the time the trials were performed [12], with the exception of tick categorisation, for which no assessment of tick engorgement was done. This is in line with the new EU [13] and WAAVP [14] guidelines and provides a sound scientific approach for systemically acting parasiticides.

To be exposed to lotilaner and other isoxazolines, parasites need to start blood-feeding. For a systemically acting product to be able to reduce the risk of parasite-borne diseases, speed of kill is therefore of major importance. There is conflicting evidence as to whether systemically acting isoxazolines could be as effective as topically acting chemicals, which may have some repellent activity, in reducing the risk of tick-borne pathogen transmission.

Transmission of pathogens such as *Borrelia*, *Anaplasma* and *Babesia* species from tick to pet hosts, is reported to typically start at least 48 hours (16 hours in some cases) after tick attachment [18–20]. Although the possibility of an earlier transmission cannot be completely excluded because of the possible release of pathogens from the ticks’ salivary glands during the process of attachment, with its speed of kill, lotilaner can contribute to reducing the risk of transmission of tick-borne pathogens.

The comparison of pre- and post-incubation data from treated cats in our study showed that, even for those ticks that are still live when removed from the animals at the earliest time-points, viability is already affected by the activity of lotilaner. This is reinforced by the observation that in the treated groups, the difference in live tick counts before and after incubation is significant at the 12 and 18 hours time-points, but not at the 24 hours assessment. This shows that ticks removed from the animals at the earliest time-points, although still alive, are already affected by the treatment and die during incubation. At the later assessment time point (i.e. 24 h), time on the animals was enough for ticks to die and incubation did not significantly impact their viability. As ticks become moribund, an impact on engorgement and transmission of pathogens cannot be excluded suggesting that, although not investigated in our study, protection against tick-borne diseases would start earlier than the actual tick death time-point. Lotilaner has, therefore, the potential to be considered as a safe and effective means of reducing the incidence of tick-borne diseases.

## Conclusions

At the minimum dose rate of 6 mg/kg, lotilaner was well tolerated and efficacious against *I. ricinus* ticks. In addition, lotilaner was effective against this tick species within 12 hours of treatment, reaching 100% efficacy within 24 hours. It sustained a rapid kill of newly infesting *I. ricinus* through 35 days. By quickly killing ticks that infest cats, lotilaner has the potential to contribute to the reduction of tick-borne pathogens transmission.

## Additional file

Additional file 1: French translation of the Abstract. (PDF 39 kb)

## Abbreviations

AE: Adverse event
ANOVA: Analysis of variance
CV: Coefficient of variation
EMEA: European Medicines Agency
EU: European Union
IVP: Investigational veterinary product
SAE: Serious adverse event
SD: Standard deviation
WAAVP: World Association for the Advancement of Veterinary Parasitology
