# Supplementary material for: Laboratory evaluation of the efficacy and speed of kill of lotilaner (CredelioTM) against Ixodes ricinus ticks on cats
Source: Parasit Vectors. 2018 Jul 13;11:413. doi: 10.1186/s13071-018-2968-4 (PMC6044019; doi:10.1186/s13071-018-2968-4)
Supplement: Supplementary file 1 — French translation of the Abstract. (PDF 39 kb) [file 13071_2018_2968_MOESM1_ESM.pdf]

Évaluation en laboratoire de l'efficacité et de la rapidité d'action du lotilaner (Credelio^{TM}) contre les tiques *Ixodes ricinus* chez le chat

Daniela Cavalleri[1*], Martin Murphy[1], Wolfgang Seewald[1], Jason Drake[2] et Steve Nanchen[1]

[1]Elanco Animal Health, Mattenstrasse 24a, CH-4058, Bâle, Suisse.
[2]Elanco Animal Health, 2500 Innovation Way, Greenfield, IN 46140, États-Unis

*Correspondance : cavalleri_daniela_a@elanco.com

Courriels des vétérinaires :
Daniela Cavalleri, cavalleri_daniela_a@elanco.com
Martin Murphy, murphy_martin_gerard@elanco.com
Wolfgang Seewald, seewald_wolfgang@elanco.com
Jason Drake, drake_jon_j@elanco.com
Steve Nanchen, nanchen_steve@elanco.com

Résumé

Contexte : Le lotilaner, autorisé chez le chien sous forme de comprimés à croquer, a été développé en parallèle en vue d'une administration orale chez le chat (comprimés à croquer pour chats Credelio^{TM}) afin de proposer un antiparasitaire pratique, sans danger et efficace rapidement. Il constitue en effet une alternative aux produits topiques fiable et pratique, tant pour les chats que pour leurs propriétaires. Ce manuscrit décrit trois études pivots de laboratoire, dont l'objectif était d'évaluer l'efficacité et la rapidité d'action du lotilaner pour tuer les tiques de l'espèce *Ixodes ricinus* chez le chat, suite à une seule administration par voie orale, à une dose d'environ 6 mg/kg.

Méthodes : Dans les études 1 et 2, l'efficacité et l'innocuité ont été évaluées sur 16 chats 48 h après le traitement initial, puis après chaque infestation hebdomadaire consécutive pendant 35 jours, par rapport à des chats témoins non traités. Dans l'étude 3, l'efficacité et l'innocuité ont été évaluées jusqu'à J35 sur 8 chats traités au lotilaner, avant et après 24 h d'incubation des tiques femelles vivantes retirées des animaux 12, 18 et 24 h après administration, puis lors des infestations hebdomadaires suivantes.

Résultats : Dans l’étude 1, l’efficacité était > 99 % à J23 et J37, et de 100 % lors de toutes les autres évaluations. Dans l’étude 2, elle était > 98% à J9 et J37, et de 100 % tous les autres jours. Dans l’étude 3, le lotilaner s’est avéré efficace à plus de 90 % à J0, pré- et post-incubation lors de toutes les évaluations. À J7, 12 heures après l’infestation, son efficacité était de 100 %, pré- et post-incubation. À J14, une réduction de 66,5 % de la moyenne géométrique du nombre de tiques vivantes a été observée chez les chats traités par rapport aux témoins, atteignant, après incubation, 94,4 %. L’efficacité a ensuite diminué en-dessous de 90 %, bien que le nombre de tiques observées au sein des groupes traités soit resté significativement inférieur à celui relevé chez les témoins. À 18 heures, le lotilaner s’est révélé efficace à ≥ 90 % jusqu’à J37, atteignant 100 % à 24 heures pour tous les jours de l’étude, sauf J28 (98,9 et 99,1 % pré- et post-incubation, respectivement). Aucun événement indésirable lié au traitement n'a été observé.
Conclusions : À une dose minimale de 6 mg/kg, le lotilaner s’est avéré efficace contre les tiques *I. ricinus*. En outre, il s'est montré efficace contre cette tique dans les 12 heures suivant le traitement et a atteint les 100 % dans les 24 heures. Une persistance de cet effet létal rapide a été observée suite aux nouvelles infestations par *I. ricinus* tout au long des 35 jours. Grâce à son action létale rapide sur les tiques infestant le chat, le lotilaner peut contribuer à réduire la transmission vectorielle de pathogènes par les tiques.
Mots clés : Lotilaner, Credelio™, chat, tique, *Ixodes ricinus*, efficacité, rapidité d’action, innocuité
